# How What We See and What We Know Influence Iconic Gesture Production

**DOI:** 10.1007/s10919-017-0261-4

**Published:** 2017-07-12

**Authors:** Ingrid Masson-Carro, Martijn Goudbeek, Emiel Krahmer

**Affiliations:** Tilburg Centre for Cognition and Communication (TiCC), Faculty of Humanities, Tilburg University, Warandelaan 2, PO Box 90153, 5000 LE Tilburg, The Netherlands

**Keywords:** Co-speech gesture, Representation technique, Iconicity, Input modality, Manipulability

## Abstract

In face-to-face communication, speakers typically integrate information acquired through different sources, including what they *see* and what they *know*, into their communicative messages. In this study, we asked how these different input sources influence the frequency and type of iconic gestures produced by speakers during a communication task, under two degrees of task complexity. Specifically, we investigated whether speakers gestured differently when they had to describe an object presented to them as an image or as a written word (input modality) and, additionally, when they were allowed to explicitly name the object or not (task complexity). Our results show that speakers produced more gestures when they attended to a picture. Further, speakers more often gesturally depicted shape information when attended to an image, and they demonstrated the function of an object more often when they attended to a word. However, when we increased the complexity of the task by forbidding speakers to name the target objects, these patterns disappeared, suggesting that speakers may have strategically adapted their use of iconic strategies to better meet the task’s goals. Our study also revealed (independent) effects of object manipulability on the type of gestures produced by speakers and, in general, it highlighted a predominance of molding and handling gestures. These gestures may reflect stronger motoric and haptic simulations, lending support to activation-based gesture production accounts.

## Introduction

Speakers often rely on *iconicity* (resemblance between form and referent) to gesturally depict attributes of referents, such as their shape or function (e.g., tracing a contour, or demonstrating the use of a tool). Despite the advancement in our understanding of how gestures are produced, we know little about the mechanisms driving the choice of such iconic strategies in spontaneous gesturing. Recently, researchers have begun to tackle this issue by studying the use of different *modes of representation* (Müller 1998) rooted in everyday habitual and artistic practices such as imitating or drawing, uncovering preferences in the way speakers manually depict objects. For instance, speakers exhibit a preference for *imitating or handling* gestures to represent objects that can be manipulated (e.g., pretending to handle a toothbrush and miming the act of brushing one’s teeth) over other suitable representation techniques such as letting the hand portray the object (e.g., using an extended index finger to represent a toothbrush, and miming the act of brushing one’s teeth) (Padden et al. 2015). Conversely, when conveying shape information, speakers tend to produce molding or sculpting gestures more often than other potentially informative gestures like tracing a silhouette (Masson-Carro et al. 2016). Regularities have also been found in how speakers choose and combine different strategies to depict objects when gestures are used in the absence of speech (van Nispen et al. 2014), highlighting convergence in the way speakers manually depict visual information. Importantly, however, the experimental research available looking at representation modes in the manual modality has mainly relied on visuospatial stimuli (pictures or video), making it hard to evaluate the extent to which the speakers’ gestural depictions reflect conceptual knowledge, or merely depict information visually present in the stimuli. If we are to understand how gestures are depictive of a speaker’s mental representation, we should examine the gestures produced when speakers are provided with a visual representation in contrast with when speakers draw only from their own conceptual knowledge. This not only helps further the discussion of how different gesture types germinate, but it also offers new insight into the nature of multimodal representation.

In this paper, we explore the effects of visually-presented stimuli (pictures) as opposed to verbally-presented stimuli (words) on gesture production about objects differing in their degree of manipulability, ranging from low (“table”) to high (“pen”). Although both are thought to access semantic memory (e.g., Caramazza 1996), pictures and words activate different (aspects of) representations in ways that are relevant for subsequent verbal and gestural representation. For instance, pictures are rich in visual detail and denote more concrete categories than words do (Amit et al. 2009), and they are likely to activate mental representations that are richer in motor content, which may influence both the frequency of gesturing and the form that gestures adopt. Furthermore, we will also examine whether the use of iconic strategies varies depending on the complexity of the descriptions speakers produce.

In the next sections, we introduce the challenges of studying gestural representation modes, and we explore the processes that may give rise to gestures when speakers draw from conceptual and perceptual knowledge.

## Background

**Fig. 1:**
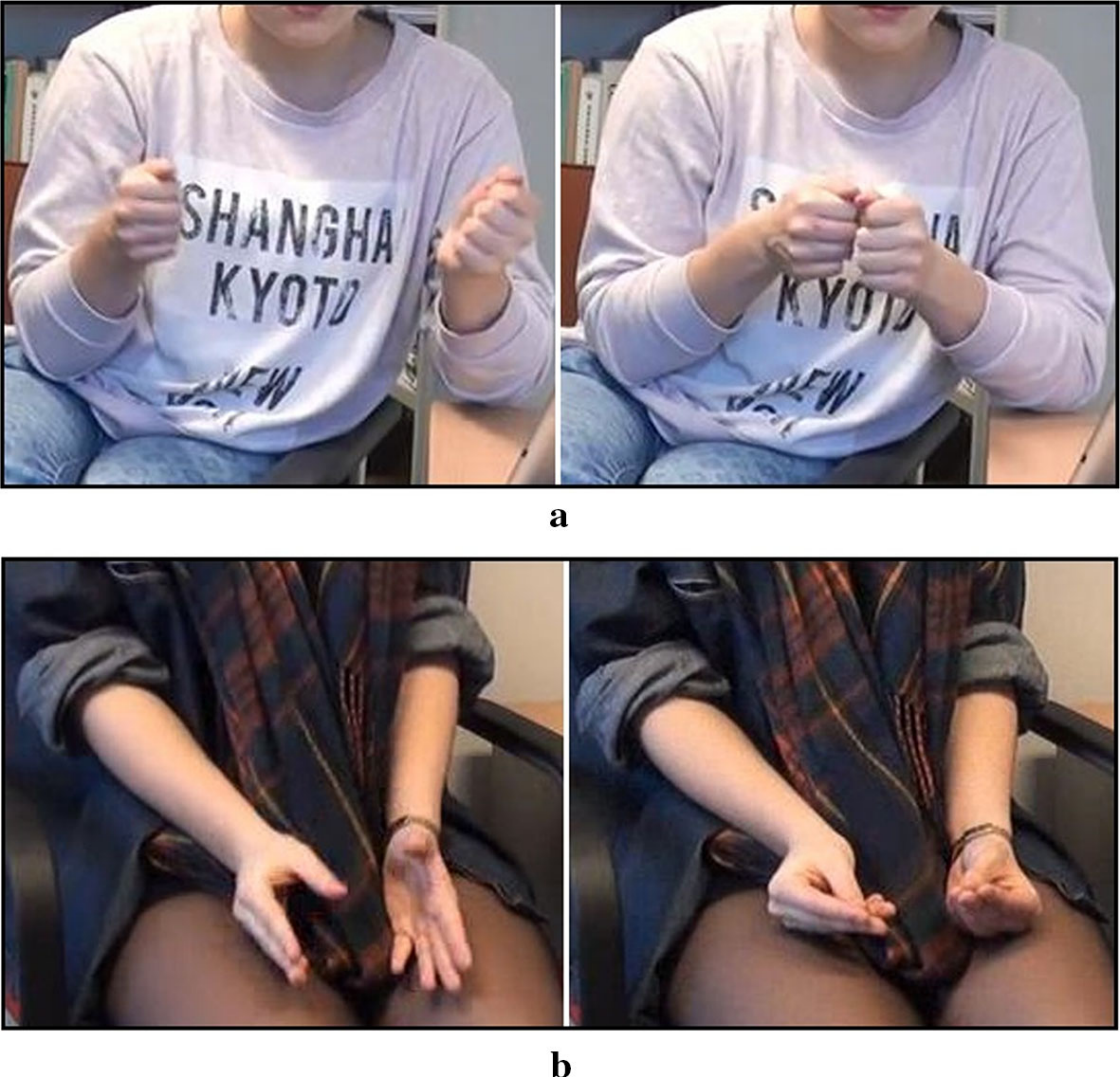
Two different gestures depicting the use of a pair of pliers, extracted from the current experiment. Speaker 1 (**a**) demonstrates the use of pliers; Speaker 2 (**b**) uses her hands to represent the object

Speakers are known for using their hands when conversing with others. Such gestures are known as co-speech gestures—as they typically occur alongside speech—and fulfill both cognitive and communicative functions (e.g., Alibali et al. 2001; Goldin-Meadow 1999). Among the various types of hand gestures (e.g., see Kendon 2004 for a comprehensive review), *iconic* gestures (McNeill 1992) depict characteristics of the referents alluded to in speech, in such a way that the gestures resemble or evoke their referents. For instance, tracing a square with one’s extended index finger creates an ephemeral image that an observer may associate with a real-world referent, say, a window or a box. Hence, these gestures receive the name “iconic” because they make use of *iconicity* (mapping between form and meaning, Emmorey 2014; Perniss and Vigliocco 2014; Taub 2001) to convey information. Despite its pervasiveness in the visual communication modality, iconicity has until recently not received much attention, deemed a more primitive form of communication in comparison with the arbitrary forms that populate speech (Tolar et al. 2008). However, there is nothing simple about how we produce and comprehend iconic signs or gestures. From the point of view of the producer, executing a visual form that is iconic of a referent may entail a series of complex processes, such as activating a suitable modal representation, identifying and selecting salient features (visual, structural, functional, etc.), and selecting^1^ an encoding strategy, all whilst taking into account the affordances of the manual modality and of the environment. The processes underlying these operations, as well as the relations amongst them, remain poorly understood, especially in co-speech gestures. In contrast with signed languages where (iconic) form-meaning mappings have become conventionalized (Perniss and Vigliocco 2014; Taub 2001), the iconicity in spontaneous gestures produced by hearing speakers may be constructed anew with each manual depiction, and may consequently express different features of a single referent each time, or even the same feature in different ways. For instance, in depicting the function of a pair of pliers, Speaker 1 (Fig. 1a) adopts a first-person perspective that allows her to demonstrate to her interlocutor how a pair of pliers is used, whereas Speaker 2 (Fig. 1b) lets her hands represent or “become” the object, her hands opening and closing symbolizing the opening and closing of the pliers’ jaws. Technically, both these iconic gestures express action, but Fig. 1a focuses on the figure of the actor and Fig. 1b focuses on the object—thus, both the perspective and the iconic strategy employed are different. Importantly, the speech produced by both speakers alludes to the same function of pliers by using similar vocabulary (“gripping things”). Where do these different strategies originate, and what causes speakers to adopt a particular strategy to represent objects and events?

### Representation Modes in Gestural Depictions

Much of the research on the use of iconic strategies in spontaneous gesturing has been inspired by the study of iconicity in signed languages (e.g., Klima and Bellugi 1979; Mandel 1977). This is unsurprising, given the common iconic basis underlying gesture and sign (Padden et al. 2015, p. 82). In the gesture domain, a few classifications of depiction techniques have been proposed, notably by Müller (1998) or Streeck (2008). Müller (1998) identifies four *representation modes* that are regularly used by gesturers to achieve iconicity. Such strategies may have naturally emerged from observing, and interacting with the world, and thus reflect habitual and artistic practices such as drawing, or sculpting. These modes comprise: *imitation*, where the speaker’s hands (and body) represent an (imaginary) character’s hands and imitate the execution of an action; *portrayal*, where the hands embody the object that they represent, such like when we extend the index and middle fingers to represent a pair of scissors; *molding*, where the hands “mold” or sculpt a shape in the air, as if palpating it; and *drawing*, where the hand traces a silhouette in the air, often with the index finger.

There are no formal proposals as to what mechanisms elicit the selection of a particular representation mode over another in spontaneous gesturing. In fact, the use of iconicity is underrepresented in most currently-available models of gesture production, which (in the interest of simplicity) only provide coarse-grained directions to how iconic gestures are generated. Most models share the assumption that gestures arise from visuospatial representations activated or generated during conceptualization (e.g., de Ruiter 2000; Hostetter and Alibali 2008; Kita 2000; Kita and Özyürek 2003). Although specific models differ in what happens next, they seem to agree that the form of gestures may be partly determined by the spatiomotoric properties of the referent or event, as well as, naturally, by the communicative intent of the speaker. One hypothesis, the gestures as simulated action framework (GSA) holds that gestures emerge from the motoric simulations that underlie the act of speaking (Hostetter and Alibali 2008), based on the premise that processing language entails sensorimotor simulation (for a review, see Fischer and Zwaan 2008). In Hostetter and Alibali’s own words, “as one moves from visual images through spatial images to motor images, the amount of action simulation increases, and, according to the GSA framework, so does the likelihood of a gesture” (p. 510). A handful of studies provide support for such a simulation account, showing, for instance, that speakers gesture more when they speak about topics high in motor content (e.g., tying one’s shoelaces), in comparison with topics eliciting mainly visual imagery (e.g., describing a beautiful landscape) and abstract topics (e.g., discussing politics) (Feyereisen and Havard 1999). Similarly, speakers gesture more when they discuss topics that are easier to generate a mental picture for (Beattie and Shovelton 2002). In addition, speakers appear to be sensitive to the *affordances* of objects (the potential for action that objects evoke; Gibson 1986), with studies showing that speakers gesture more when describing highly manipulable objects (e.g., a comb) than less manipulable objects (e.g., a table) (Hostetter 2014; Masson-Carro et al. 2016; Pine et al. 2010). These affordance effects have also been observed in “co-thought” gestures, for instance when speakers solve a spatial task in silence (Chu and Kita 2016), suggesting that gestures can be generated directly from action simulations and independently of speech.

If gestures are the physical outcome of imagery simulations, we could expect gestures to inherit aspects of such simulations. In the case of motor simulations, it seems reasonable to assume that gestures will resemble everyday actions. For other types of imagery simulation (e.g., visual imagery) the connection may seem less obvious, but it could be that speakers simulate sensorial patterns associated to objects, for instance their touch, or their weight on one’s hands as objects are moved or transported, and that this facilitates the transduction of a mental image into overt movement. A few studies yield insight into the use of iconic strategies in spontaneous gesturing. It appears that factors such as one’s own motoric experience (Cook and Tanenhaus 2009), the characteristics of the referent (Masson-Carro et al. 2016), or even the input modality (pictures or video) (Padden et al. 2015) can have a say in what gestural encoding strategies will be employed by speakers.

For example, Cook and Tanenhaus (2009) had speakers explain the solution to the Tower of Hanoi task after having solved it using real objects, or on a computer, using the mouse pointer. Speakers in the real-action condition more often used grasping gestures while explaining the solution, but their computer counterparts more often drew the trajectory of the mouse as it appeared on the screen during the accomplishment of the task. Thus, the gestural techniques used did more than just explaining the task, they gave specific insight into the speaker’s own motoric experience. Similar effects have been found in narration retelling tasks involving a character in a story. Parrill (2010) showed that speakers more frequently adopt a first-person perspective (e.g., miming the performance of the actions that the character performs) when the character’s hands or torso are prominent in the original story—a possible case for *mimicry* effects (Chartrand and Bargh 1999). In contrast, speakers were more likely to depict actions from a third-person perspective (e.g., tracing the path along which the character moved) if the emphasis was not placed on the figure of the character.

Masson-Carro and colleagues (2016) analyzed the representation modes observed in gestures accompanying descriptions of objects, and found that manipulable objects predominantly elicited imitation gestures, whereas less manipulable objects predominantly elicited shape representations. Moreover, their study highlighted the predominance of two particular modes, namely (transitive) *imitation* (referred to as *object use*, p. 440) and *molding* gestures. This preference towards techniques that closer depict haptic knowledge is in line with embodiment accounts of gesture production and illustrates the claim that some iconic types stem from simulations of acting on objects (as is the case for imitation) and *exploratory practices* (as is the case for molding) such as contour following or enclosing, as proposed by Lederman and Klatzky (1987). Such preferences appear to be modulated by the modality of presentation. A study by Padden and colleagues (2015) compared the use of two representation modes to depict man-made tools, namely imitation (which they call *handling*) and portrayal (which they call *instrument*) in adult hearing speakers and deaf signers. They show that hearing speakers have a preference for imitating techniques to represent manipulable objects (e.g., pretending to handle a toothbrush and miming the act of brushing one’s teeth) in contrast to letting their hand portray the object (e.g., using an extended index finger to represent a toothbrush). Interestingly, their study featured two conditions, pictures and video, and although hearing speakers produced more imitating gestures in both conditions, portraying gestures occurred more often in the pictorial condition than in the video condition, and the opposite was true for imitating gestures. This study hints the importance of assessing the issue of representation by looking at different presentation modalities.

Van Nispen and colleagues (2014) assessed the use of representation techniques in *pantomime* (McNeill 1992), more recently called *silent gesture* (e.g., Özçalışkan et al. 2016), and found some regularities regarding what techniques were chosen to pantomime objects across participants, and also how they were combined, which led them to hypothesize that speakers may share *intrinsically similar mental representations* about objects (p. 3024). A recent study by Özçalışkan et al. (2016) supports this idea by showing that when speech is present, the way gestures concatenate to depict an event is constrained by the concurrent speech (an idea first put forth by Kita and Özyürek 2003), but when gestures stand on their own, a natural order of events comparable to SOV patterns (subject–object–verb) emerges.

### Visual or Verbal Input Modality: Implications for Gesture Production

It becomes clear that representation modes are not just ways to convey information to an interlocutor, but that they hold the potential to reveal information about how speakers conceptualize objects and events. One key aspect that, in our opinion, has been overlooked, is the fact that most studies examine speakers’ gestures after being exposed to visual stimuli. This implies that speakers have been provided with a concrete visual representation on which to base their gestural depiction, either online (whilst seeing it) or from memory. It is only natural to assume that a speaker’s gestures may look different if describing a pair of scissors based on a picture than on a word. Processing pictures compared to words may lead to the activation of different representations, and may guide the aspects of a representation that speakers will pay attention to, which should affect the type of gestures produced. Extensive research from an embodied cognition perspective has shown that perceiving objects with a strong action component, whether in a visual (pictorial, video) or verbal form (written, audible), recruits motor processing (Borghi 2015). From a gesture perspective, however, there are reasons why stimuli input modality might alter gestural output. First of all, we do not know the extent to which the motor evocation caused by words is the same as that caused by pictures (Bub and Masson 2006, p. 1113), something that could influence the frequency as well as the form of gestures—following Hostetter and Alibali’s (2008) predictions. Secondly, although both pictures and words access semantic knowledge (Caramazza 1996; Vandenberghe et al. 1996), they are in essence different representations, and even when referring to a concrete entity known by the speaker (e.g., the mug where someone drinks coffee from every morning), attending to each may confer saliency to different aspects of the same object. At a basic level, pictures are rich in visual detail and evoke more concrete categories than words do (Amit et al. 2009). One could hypothesize that richer (visual) representations may activate stronger simulations of perceptual and motoric content and thus give rise to more gesturing—and more gestures depicting action—because they highlight the potential for action. Further, it could be that pictures direct the speaker’s attention toward perceptual aspects of representations (like shape, size, or color), and words instead activate accessible and less variable attributes, such as function. In other words, it could be that when speakers attend to a picture, they will tend to produce gestures that depict perceptual aspects of a referent (e.g., shape) and when they attend to a word, they may be more likely to talk—and gesture—about function.

Few studies have examined the effects of stimuli presented as images (video, in these cases) or text,^2^ on gestures. These studies have focused on narrative retellings from memory (Hostetter and Hopkins 2002; Hostetter and Skirving 2011; Parrill et al. 2010), with inconclusive results. Two of these studies (Hostetter and Hopkins 2002; Hostetter and Skirving 2011) showed that speakers produced more iconic gestures while retelling a cartoon they had viewed, as opposed to after reading its description. The authors conclude that having access to a richer visual representation may have boosted stronger simulations of mental imagery, leading to an increase in gesturing. The content of gestures, though, was left unassessed in both studies, so it remains an open question whether speakers used similar depicting strategies across experimental conditions. Parrill et al. (2010) did not find differences in terms of gesturing frequency between the visual and verbal conditions. This study also examined gestural viewpoint but did not find speakers to produce more first-person viewpoint gestures (e.g., imitative gestures) in the video condition. One hypothesis as to why no effects were found is that in the textual condition speakers may have tried to visualize the content of speech more consciously in order to construct a good retelling for their addressee, which could have overridden a possible condition effect. This suggests that the effects of input modality on gesturing may vary depending on methodological factors related to, for instance, the effort put into a task. We find this makes an interesting aspect to investigate, from both a theoretical and a methodological standpoint.

In this study, we operationalize task effort by having a condition where speakers produce simple descriptions by allowing them to name the objects during the task, and a condition where speakers produce more elaborate descriptions, by prohibiting them to name the objects. It is often the case that experimental paradigms forbid speakers to explicitly name (parts of) the target objects in communication tasks (e.g., Hostetter 2014; Pine et al. 2010) but, while this is a valid approach, it is more often the case that speakers in real-life conversation know and can verbalize what they want to say, than not. Therefore, this manipulation will offer insight into whether speakers still produce gestures—and what kind of gestures—when they are given the choice to simply read out loud the names of the objects.

### The Present Study

This study examines the effects of visual (pictures) and verbal stimuli (words) on gesture production about objects differing in functional manipulability in a referential task. Furthermore, we examine these effects under two levels of describing complexity, by allowing or forbidding speakers to name the objects during the task.

In order to gain insight into how input modality influences gesture production, we will annotate (1) the occurrence of gestures, and (2) the representation mode most predominantly associated to each gesture. In contrast to previous similar research (e.g., Hostetter and Skirving 2011; Parrill et al. 2010), our visual stimuli consist of pictures of real-world objects, and not videos featuring moving entities or cartoons, and the stimuli will remain visible to the speakers during the task. In this way, our manipulation allows to examine representation techniques unconstrained by (1) salient narrative events, and by (2) possible mimicry effects stemming from watching a character move and act, all while keeping the speaker’s memory demands to a minimum. In sum, we hypothesize that speakers will gesture more when they attend to images than when they attend to words. We also expect the presentation modality to guide what aspects of an object will be represented in gesture, with pictures giving rise to more perception-based iconic strategies (e.g., molding or tracing gestures) and words leading to more action-based iconicity (e.g., pretending to handle an object).

## Method

### Design

Pairs of participants were recorded during a referential task in which speakers described a series of items to a listener, who subsequently accomplished a memory test. The experiment followed a mixed 3 × 2 × 2 design, with manipulability degree as within-subjects (high manipulability, low manipulability, and animals), and stimuli presentation (verbal and visual), and naming (allowed and forbidden), as the between-subjects variables.

### Participants

Ninety undergraduate students from Tilburg University took part in this experiment, in exchange for course credit points. All participants were native speakers of Dutch, and carried out the experimental task in pairs where one was the speaker and the other was the listener, therefore we collected descriptions from forty-five speakers (*M* = 20.8; *SD* = 2.5; 29 female).

### Stimuli

**Fig. 2:**
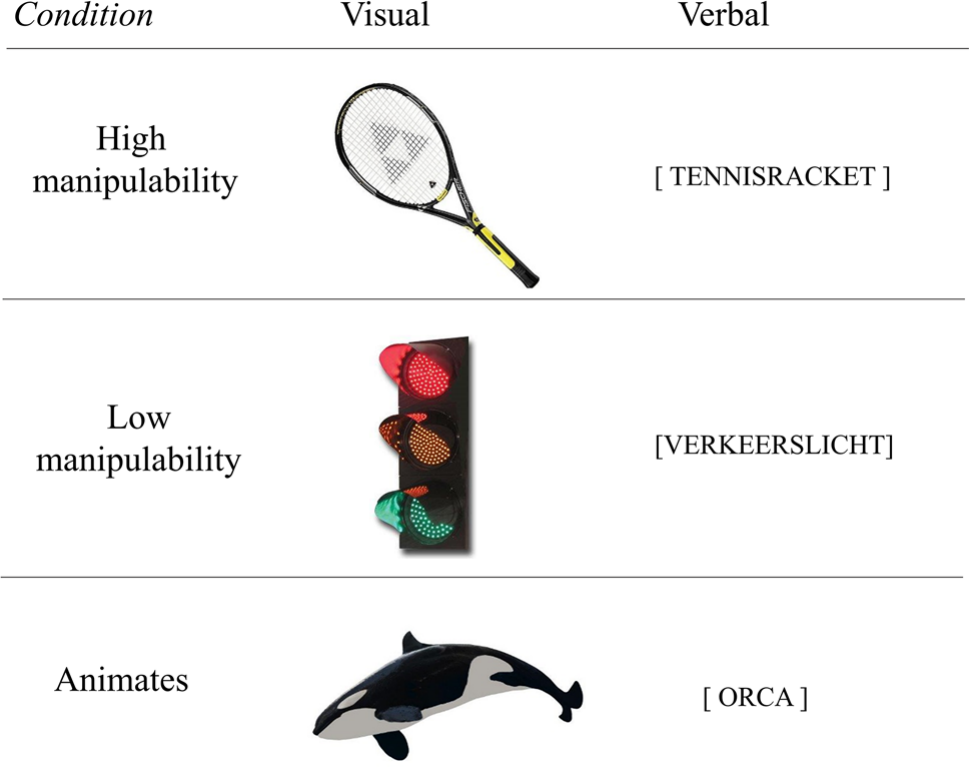
Three example items from the stimuli dataset, displayed both in their pictorial form (*middle column*), as well as in their verbal form (*right column*)

The stimuli consisted of pictures and written words denoting objects that differed in manipulability, namely high-manipulability objects (e.g., toothbrush, comb), low-manipulability objects (e.g., cross, castle), and animals (e.g., giraffe, elephant) (Fig. 2). We define highly manipulable objects as *objects operated exclusively with the hands, whose operation may induce a change in the* (*physical*) *world* (Masson-Carro et al. 2016). For instance, using a pen to write a sentence leaves a physical trace on a surface, provided that the object is used for its conceived purpose. On the contrary, the function associated with low-manipulability objects is not primarily executed by the human hands. For instance, we sit at a table to perform several actions (e.g., eating, writing), but none of these actions is typically carried out by direct, physical manipulation of the table itself. We included animals as a control condition, given that animals are animate entities, and according to our definition of manipulability they cannot be manipulated.

One hundred images were selected from the Bank of Standardized Stimuli (BOSS, Brodeur et al. 2014), a freely accessible dataset of visual stimuli. Although the stimuli in BOSS have been thoroughly standardized for several variables, including naming and manipulability, we conducted an additional pre-test to ensure that both the pictures and the corresponding words denoting them were perceived similarly (see below) by a Dutch-speaking audience. We administered the pre-test using CrowdFlower (an online crowdsourcing service; http://www.crowdflower.com) to 62 native speakers of Dutch (*M* = 42.3; *SD* = 13.1), who were randomly assigned to one of two conditions: verbal or visual.

In the visual condition, participants were first asked to name (in Dutch) the objects that were displayed on their screen. We did this to ensure (1) that the names we had assigned to the objects matched those assigned by the majority of the participants, and (2) that the objects were easy to name, to avoid possible effects of verbal encodability on gesture production (e.g., Morsella and Krauss 2004). Secondly, participants rated the manipulability of the objects displayed on a scale from 0 to 100 (with 0 being the least manipulable and 100 the most). The definition of manipulability was adapted from Hostetter (2014, p. 1472: *when I imagine this object, I imagine touching it, holding it, or using it in a specific way, and the way the item would be used is one of its most dominant features*).

In the verbal condition, participants rated the words (denoting the same objects than the pictures did in the visual condition) for manipulability following identical guidelines, and in addition they had to indicate if the words were easily imageable (i.e., when I imagine this word, it is easy for me to visualize the object it corresponds to; yes/no).

Our criteria for stimuli inclusion were strict. First, all pictorial items where naming agreement was low (<80%) or did not match the name we had assigned to the objects in the verbal condition were excluded. For the verbal condition, we excluded all items whose imageability scores lay under 90%. This led to the selection of 49 objects (49 pictures and their corresponding 49 verbal affiliates): 19 highly manipulable, 17 low manipulable, and 13 animals. Thus, the stimuli were both easily nameable (*M* = 90.35, *SD* = 9.3) and highly imageable (*M* = 96.26, *SD* = 4).

Next, we computed the joint manipulability scores (word-based and picture-based) for all items. For highly manipulable objects, all items whose joint manipulability rating was below 90% were removed. The manipulability scores for low-manipulability objects ranged from 20 to 50%, and for animals the scores ranged between 12 and 30%. Importantly, the scores in each object category were very similar in the visual and verbal conditions. Table 3 in Appendix 1 shows an overview of the scores for each of the items used in the study.

The stimuli items for the task were compiled into a presentation document, where high- and low-manipulability objects, and animals, were interspersed. Each item was presented in a separate slide, therefore the speaker saw (and described) one object at a time, and moved forward through the items by pressing the spacebar on the laptop’s keyboard at the end of each description. The interspersion of the stimuli was achieved by generating a semi-randomized list with all items, which we then used as the presentation order (A). We ensured that same-type items would not repeatedly occur consecutively (e.g., three animals in a row). We created a second presentation list (B) where the items’ order was counterbalanced.

### Procedure

**Fig. 3:**
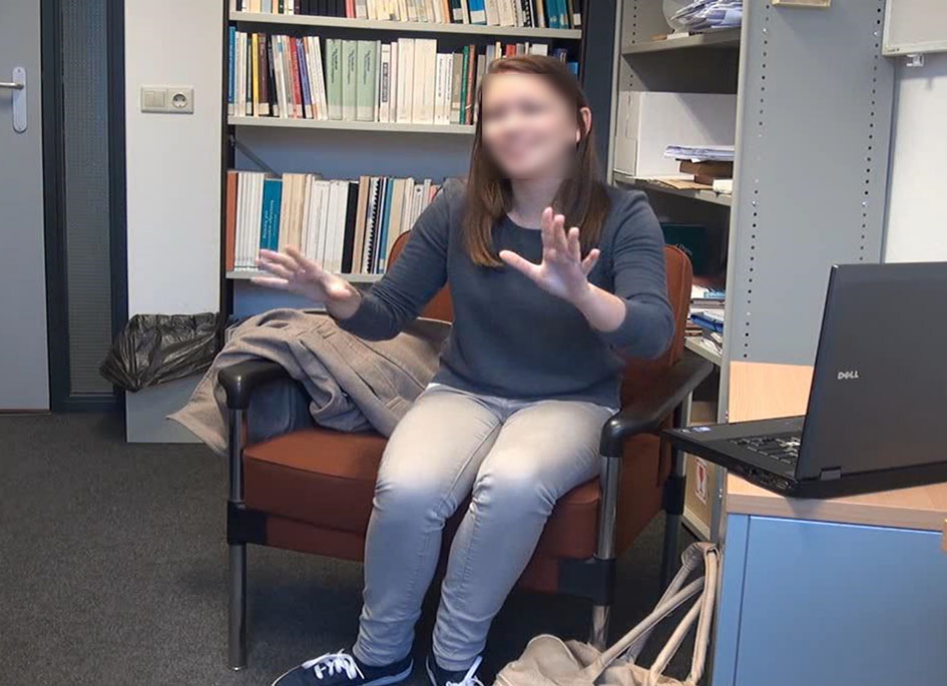
Example of the set-up, as captured by the camera videotaping the speaker. The image shows the speaker describing objects to the listener, who is located exactly in front. To the left of the speaker, the stimuli are displayed on a 15″ laptop

Upon arrival to the experiment room, participants were assigned the roles of speaker and listener in order of arrival (the first participant to arrive to the experimental room was assigned the role of speaker), and sat opposite each other in comfortable seats to facilitate their engagement in the interaction. To the left of the speaker, a low table was placed with a 15″ laptop on top displaying the task’s stimuli, in such way that only the speaker, but not the listener, would see the content of the screen (Fig. 3). A camera was positioned beside the listener, with the goal of recording the speaker’s full body movements. The experiment was conducted in three separate rooms due to availability issues, but the experimental set-up was kept identical for all participant pairs, with minimal variation in the camera angles.

Table 1 shows the order followed to assign pairs to the experimental conditions, which was done prior to the commencement of the testing phase. Crucially, half of the participants (23) saw pictures (visual condition) and 22 saw words (verbal condition). We also included naming as a between-subjects variable in our design, with 19 speakers being allowed to name the objects that had to be described, and 26 speakers being banned from doing so. The difference in number of participants for the naming allowed and forbidden conditions stems from unplanned dropout of participants.

**Table 1:** Template used to assign pairs of participants to the experimental conditions

Pair	Condition	Presentation order
Pair 1	Verbal—Allowed	A
Pair 2	Verbal—Forbidden	A
Pair 3	Visual—Allowed	A
Pair 4	Visual—Forbidden	A
Pair 5	Verbal—Allowed	B
Pair 6	Verbal—Forbidden	B
Pair 7	Visual—Allowed	B
Pair 8	Visual—Forbidden	B
Pair 9	Verbal—Allowed	A
(et cetera)	…	…

Before beginning the task, both participants read and signed the corresponding consent forms, and received written instructions regarding their role in the experiment. Each pair first completed one practice trial, consisting of the description of one item. The experimenter was present during the practice trial, and answered the participants’ questions, if there were any.

The experiment was straightforward: the participant who was assigned the role of speaker had to describe, one by one, a series of 50 items (49 target items, plus the training item) to the listener, who afterwards completed a memory test assessing the number of items she remembered correctly. Hence, participants thought that they were taking part in a memory experiment. This way, we ensured that speaker and addressee paid close attention to each other, instead of having a speaker produce descriptions towards an addressee who is busy performing a matching task.

No specific guidelines were given as to how the objects should be described, aside from the instruction prohibiting speakers to name the items in the no-naming condition. The instructions were identical in all conditions, and simply advised the speaker to describe each item as efficiently (but informatively) as possible. We expected that speakers might just name the objects when naming of the objects was allowed, but although they sometimes did, their descriptions often provided information about additional attributes (e.g., *a lamppost, which emits light during the night; a lion is a dangerous, big animal; a shed, to store stuff*; *an ant, small insect*). “Appendix 2” shows the task instructions as received by the speakers in all four conditions, translated from Dutch to English. Neither the instructions nor the experimenter made any allusion to the use of gestures.

During the description task, the listener was instructed to pay close attention to the speakers and to signal understanding of the items described. The listener could ask the speaker (clarification) questions at any time, thereby prompting dialogical interaction. After the description task ended, the speaker left the room, and the listener performed a recognition test on the computer. In this test, 100 verbal items (50 of them corresponding to the items described) were presented to the speaker in columns. The task of the listener was to drag and drop all the items she believed the speaker to have described into a box located on the right side of the screen. This test lasted approximately 5 min.

### Data Analyses

The data annotation was performed using the multimodal annotation tool Elan (Max Planck Institute for Psycholinguistics, Nijmegen, The Netherlands, http://www.lat-mpi.eu/tools/elan; Wittenburg et al. 2006). We marked the beginning of a description the moment when a speaker pressed the button that started a trial (trial = the description of a new object), and the end when the speaker concluded her description, or was interrupted by the addressee (e.g., feedback, clarification request, etc.). We then annotated all the speech and gestures for each description. Speech was transcribed verbatim, using a code word for hesitations (*hm…, ehh…*) so that they could be subtracted when computing the gesture rate (see below).

#### Gesture Annotation

We annotated each occurring hand gesture in its full length (from preparation to retraction, McNeill 1992), and classified it as iconic or non-iconic. Iconic gestures were defined as movements of the hands and arms that conveyed information related to the objects referred to in the concurrent speech, for instance by describing physical qualities or actions related to such objects. An example of an iconic gesture is tracing a circular shape with the finger whilst talking of a basketball, or mimicking the use of an instrument or tool (as in Fig. 1). Non-iconic gestures mostly comprised biphasic movements used to emphasize words (*beats*, McNeill 1992) and interactive gestures directed at the addressee (Bavelas et al. 1992; Kendon 2004). Adaptors (Ekman and Friesen 1969) and other irregular movements such as, self-scratching, or posture shifts were excluded from our annotation.

**Fig. 4:**
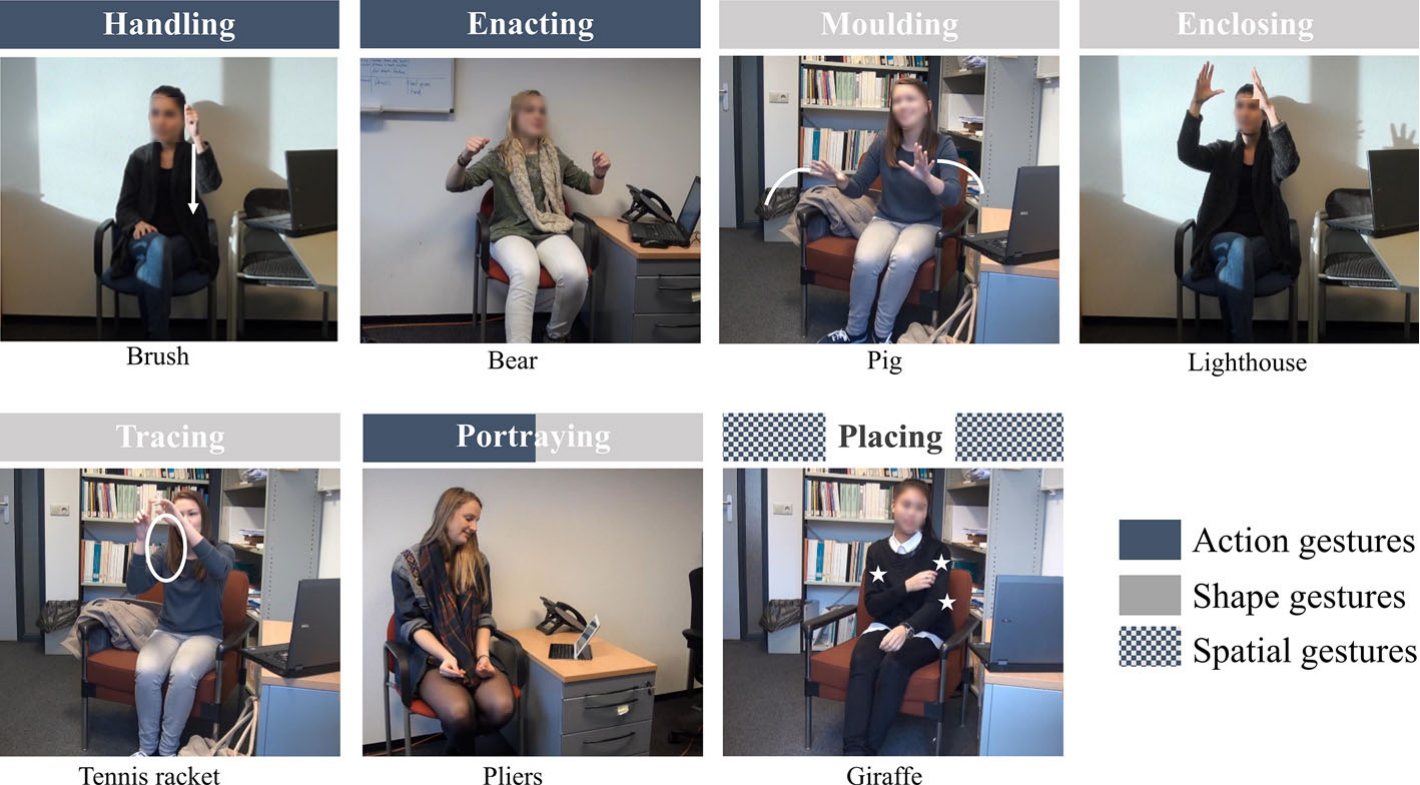
Examples of the representation techniques annotated in the present study

Descriptions varied in terms of the number of words uttered. Therefore, in order to calculate gesture frequencies under our different experimental conditions, we computed a normalized gesture rate to account for the variability in speech. This gesture rate was computed by dividing the number of gestures by the number of words for each description, and multiplying the product by 100. In addition, we annotated all iconic gestures for representation technique. Our coding scheme consisted of 7 categories stemming from Müller’s (1998) basic representation modes, and expanded based on Masson-Carro et al. (2016), and on our observations after screening the first videos of the dataset. Below are our categories (see also Fig. 4), linked to Müller’s representation modes:

1. *Imitation* where the speaker’s hands (and body) represent an (imaginary) character’s hands and imitate the execution of an action. This representation mode may be subdivided into transitive and intransitive action, depending on whether the action imitated involves an imaginary object or not. We refer to the former as *handling* gestures (term also used by Streeck 2008; van Nispen et al. 2014), and to the latter as *enactments*.
2. *Portrayal* where the hands embody the object that they represent, such like when a speaker extends the index finger as if it were a toothbrush (and may proceed to mimic the action of brushing one’s teeth). We refer to these as *portraying* gestures.
3. *Molding* where the hands “mold” or sculpt a shape in the air, as if palpating it. We separate (dynamic) *molding* gestures from (static) *enclosing* gestures, the latter referring to the hands enclosing a shape in a static way.
4. *Drawing* where the hand traces a silhouette in the air, often with the index finger. These gestures also depict shape, but they do so more schematically than molding gestures, the result—if captured in the air—resembling a two-dimensional blueprint. We refer to this category as *tracing* gestures.
5. *Placing* (see, e.g. Bergmann and Kopp 2009; Masson-Carro et al. 2016) was added to the coding scheme to account for gestures that relate to the physical structure or distribution of objects. Thus, when *placing*, the hand anchors or places an entity within the gesture space, or explicitly expresses the spatial relation between two or more entities. For instance, in Fig. 4, the speaker describes the pattern on a giraffe’s skin, and produces a sequence of strokes to place several dots on her own body.

#### Interrater Agreement

The complete dataset was annotated by the main author. In order to validate the appropriateness of the coding scheme employed, a second rater (a researcher experienced in the annotation of gestures and of representation techniques but unaware of the experimental conditions or of the aim of this particular study) annotated the first three videos (147 descriptions) based on the coding scheme above. A Cohen’s Kappa test (Cohen 1968) revealed substantial agreement for the annotation of representation techniques (κ = 0.73, *p* < .001); and a weighted Kappa test—suited for ordinal variables—showed very good agreement for gesture frequency (κ*w* = 0.93, *p* < .001), based on the number of gestures identified by each rater for each description.

#### Statistical Analyses

Our statistical procedure relied on linear mixed models for continuous dependent variables (i.e., gesture rate; number of words), and logit mixed models for categorical dependent variables (i.e. representation techniques) (see Jaeger 2008). In all of the analyses, the fixed factors were *modality* (verbal, visual), *manipulability* (high manipulability, low manipulability, animals) and *naming* (allowed, forbidden), and *participants* and *items* (stimuli objects) were included as random factors. We started with a full random effects model (following Barr et al. 2013), and eliminated the random slopes with the lowest variance in case the model did not converge.

## Results

### Gesturing Frequency

**Fig. 5:**
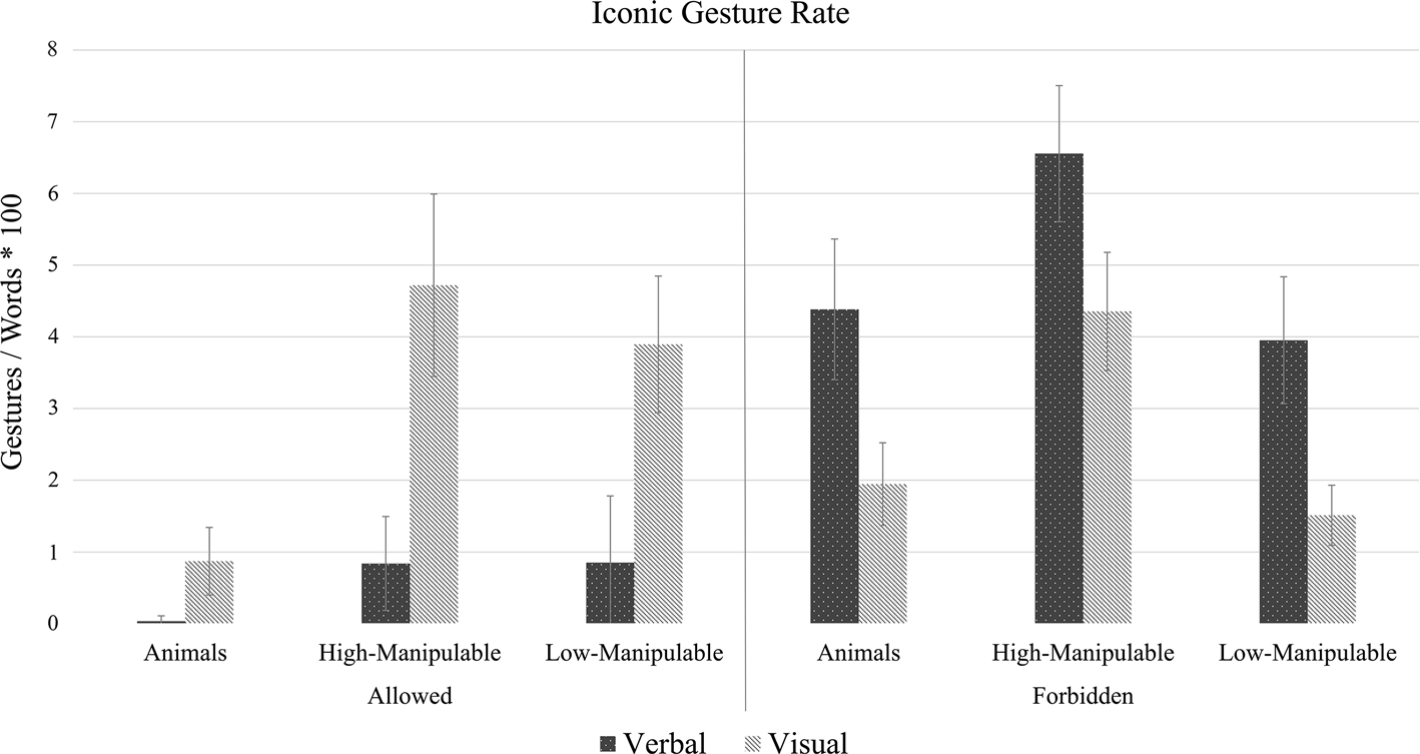
Gesture rate means for iconic gestures as a function of manipulability, modality, and naming. The *error bars* indicate (95%) confidence intervals

A total of 2067 gestures were produced during the task, of which 1188 were iconic and 879 were non-iconic. Our first aim was to investigate whether attending to pictures or to words—about objects differing in their degree of perceived manipulability—affected the frequency of gesturing. Figure 5 shows the iconic gesture rate means and confidence intervals for all experimental conditions, and Table 2 presents descriptive statistics for all variables analyzed. We first review the main effects of *manipulability* and *naming* on gesturing frequency, to then focus on the effects of *modality* (visual, verbal) and all noteworthy interactions.

**Table 2:** Means and standard deviations (SD) for the frequency of iconic and non-iconic gestures, and for the number of words per description, under each of the experimental conditions

	Allowed		Forbidden	
	Verbal	Visual	Verbal	Visual
Iconic gesture rate				
High-manipulable	.83 (4.36)	4.71 (8.9)	6.55 (7.59)	4.35 (6.58)
Low-manipulable	.85 (5.85)	3.89 (6.34)	3.95 (6.7)	1.51 (3.17)
Animals	.05 (.4)	.87 (2.7)	4.38 (6.5)	1.94 (3.8)
Non-iconic gest. rate				
High-manipulable	.92 (3.1)	1.67 (4.79)	3.21 (5.78)	2.8 (5.6)
Low-manipulable	1.2 (3.49)	2.68 (6.76)	3.39 (6.6)	2.97 (6.01)
Animals	.89 (3.39)	1.03 (3.83)	2.52 (5.76)	1.93 (4)
Words/description				
High-manipulable	10.1 (7.78)	10.78 (11.79)	20.11 (16.12)	17.09 (11.03)
Low-manipulable	10.6 (8.2)	16.46 (16.51)	19.02 (12.93)	20.43 (11.55)
Animals	9.36 (7.57)	8.57 (8.93)	18.17 (12.78)	22.11 (15.48)

We found a strong effect of *manipulability* on the iconic gesture rate, indicating that speakers gestured more when describing highly manipulable objects than less manipulable objects (β = 1.75, *SE* = 0.39, *t* = 4.47, *p* < .001), and animals (β = 2.34, *SE* = 0.42, *t* = 5.55, *p* < .001). This effect was restricted to the production of iconic gestures, which means that we found no differences for non-iconic gestures about different object types (β = 0.59, *SE* = 0.3, *t* = 1.94, *p* = .13; β = −0.39, *SE* = 0.28, *t* = 1.36, *p* = .36), and for the number of words uttered per description (β = −1.75, *SE* = 0.39, *t* = .12, *p* = .99; β = −2.03, *SE* = 1.3, *t* = −1.55, *p* = .27).

Next, there was a main effect of *naming*, indicating that speakers produced more gestures when they were not allowed to name the objects during the task—whether these gestures were iconic (β = 1.8, *SE* = 0.82, *t* = 2.2, *p* = .03) or non-iconic (β = 1.4, *SE* = 0.65, *t* = 2.16, *p* = .03). Naturally, speakers also spoke more when they could not name the objects (β = 8.23, *SE* = 1.83, *t* = 4.48, *p* < .001), but it must be noted that the results regarding gesture rates do not merely result from participants speaking more in the naming-forbidden condition, as our gesture rate measures are already averaged by the number of words spoken per description to avoid such confound.

**Fig. 6:**
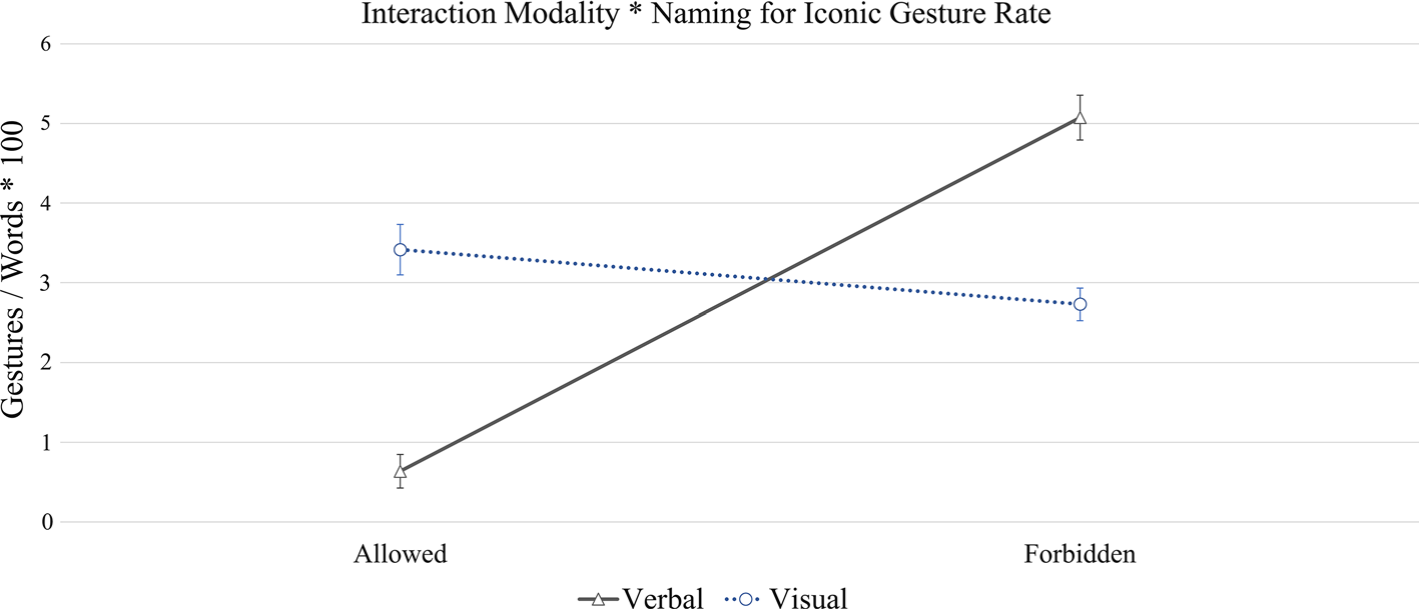
Interaction effect between *modality* and *naming*, for iconic gesture rate. The *error bars* indicate the standard error

There was no main effect of stimulus *modality* on iconic gesture rate (β = −1.9, *SE* = 0.81, *t* = −.23, *p* = .81), non-iconic gesture rate (β = .08, *SE* = 0.64, *t* = −.13, *p* = .89) or the number of words produced (β = 1.1, *SE* = 1.81, *t* = .68, *p* = .54), which means that speakers spoke and gestured to similar extents when they described a picture or a word. Nevertheless, we found a crossover interaction between modality and naming (*p* = .001) that reveals an interesting, yet somewhat unexpected picture (Fig. 6). Speakers did produce more gestures in the visual than in the verbal condition, as we had initially hypothesized, but this only happened when naming was allowed. When we forbade speakers to name the objects, speakers produced more gestures in the verbal than in the visual condition. Importantly, this pattern arises only for iconic gestures, and is not observed in either non-iconic gestures (*p* = .33) or in the number of words uttered per description (*p* = .76).

#### Manipulation Check

The names of the objects that were described in this study differed in terms of their complexity (e.g., simple nouns like “owl” and compounds such like “toothbrush”) and of their length (e.g., “ant”, 3 letters; “screwdriver”, 11 letters). We ran a manipulation check to ensure this did not have an effect on the number of gestures produced by speakers. For instance, it remains an open debate how people process compounds (Semenza and Luzziatti 2014), so we cannot discard the possibility that the constituents in a word such as “screwdriver” are processed serially, or even that they activate simultaneous representations, something which could increase gesturing. The results showed that word complexity (2 levels, simple, and compound) did not have an effect on the number of gestures produced by speakers (β = −.52, *SE* = 0.41, *t* = −1.27, *p* = .21) and the full model did not show significant differences when including or excluding word complexity as a factor (*p* = .12). Likewise, the correlation between the number of letters that words had and the number of gestures produced by speakers vas very weak, *r*(2194) = .12, *p* < .001.

### Representation Techniques’ Analyses

Our second aim was to investigate whether attending to pictures or to words affected the strategies that speakers employed to construct meaning with their hands. In general (all conditions collapsed), molding (*M* = .31, *SD* = .46) and handling (*M* = .29, *SD* = .45) were the most frequently used representation techniques to represent objects, followed by tracing (*M* = .11, *SD* = .31), enclosing (*M* = .11, *SD* = .31), portraying (*M* = .06, *SD* = .25), placing (*M* = .05, *SD* = .22), and enacting (*M* = .02, *SD* = .14). Our analyses reveal a main effect of manipulability on nearly every representation technique, and again a series of interactions between modality and naming, which co-influenced the representation techniques used by speakers independently of the manipulability degree of objects. All means and standard deviations can be found in Table 4 of Appendix 3.

**Fig. 7:**
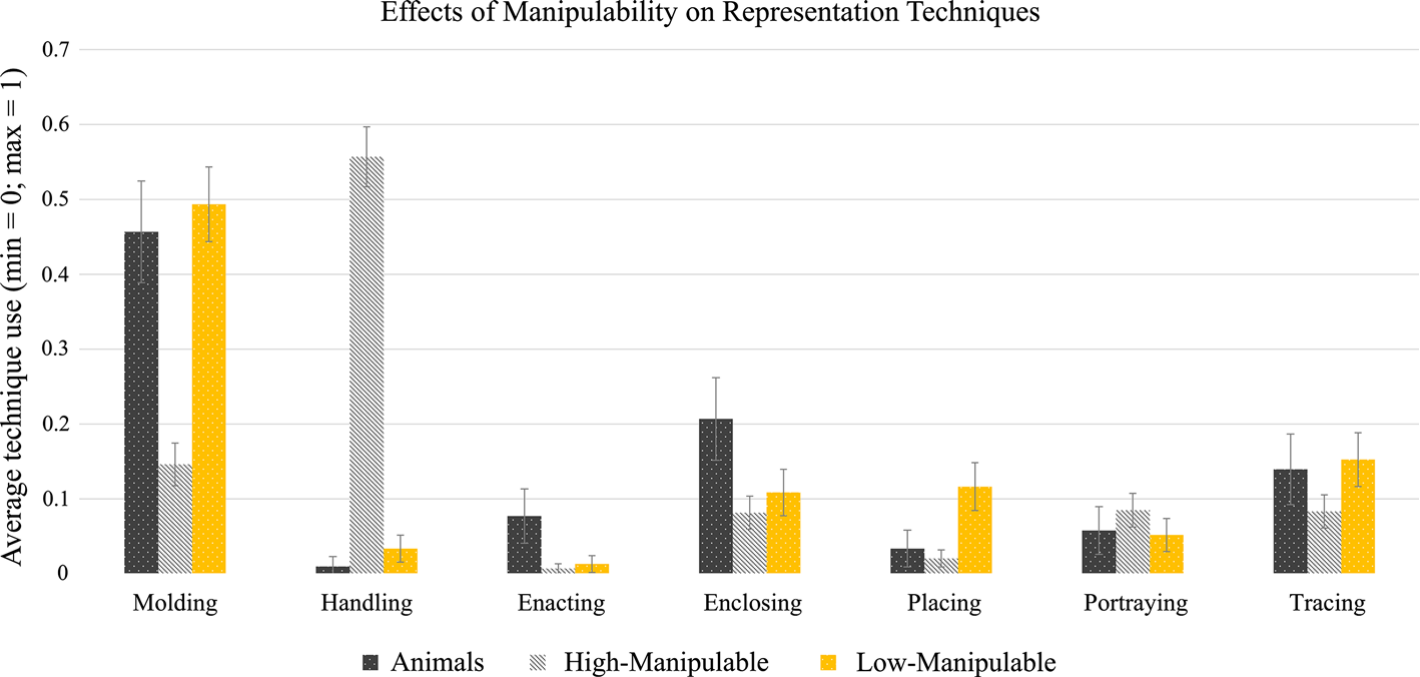
Mean representation technique frequency, as a function of manipulability. The *error bars* indicate (95%) confidence intervals

*Manipulability* determined what techniques speakers use to communicate about objects, as can be seen in Fig. 7. When speakers described highly manipulable objects, they produced mostly handling gestures, in comparison with less manipulable objects (β = 3.68, *SE* = .36, *p* < .001) and animals (β = 5.09, *SE* = .75, *p* < .001). In contrast, they produced relatively few molding gestures, in comparison with when they described less manipulable objects (β = −1.74, *SE* = .28, *p* < .001) and animals (β = −1.82, *SE* = .31, *p* < .001), as well as fewer tracing gestures as compared with animals (β = −.79, SE = .37, *p* = .03).

Enacting and enclosing gestures were produced more often when describing animals than when describing highly manipulable (β = 2.44, *SE* = .56, *p* < .001; β = 1.02, *SE* = .22, *p* < .001—respectively) and less manipulable objects (β = 1.66, *SE* = .52, *p* = .004; β = .58, *SE* = .24, *p* = .04—respectively). In addition, speakers produced placing gestures mostly to describe low-manipulability objects (β = −1.33, *SE* = .56, *p* = .04; β = −1.93, *SE* = .48, *p* < .001).

**Fig. 8:**
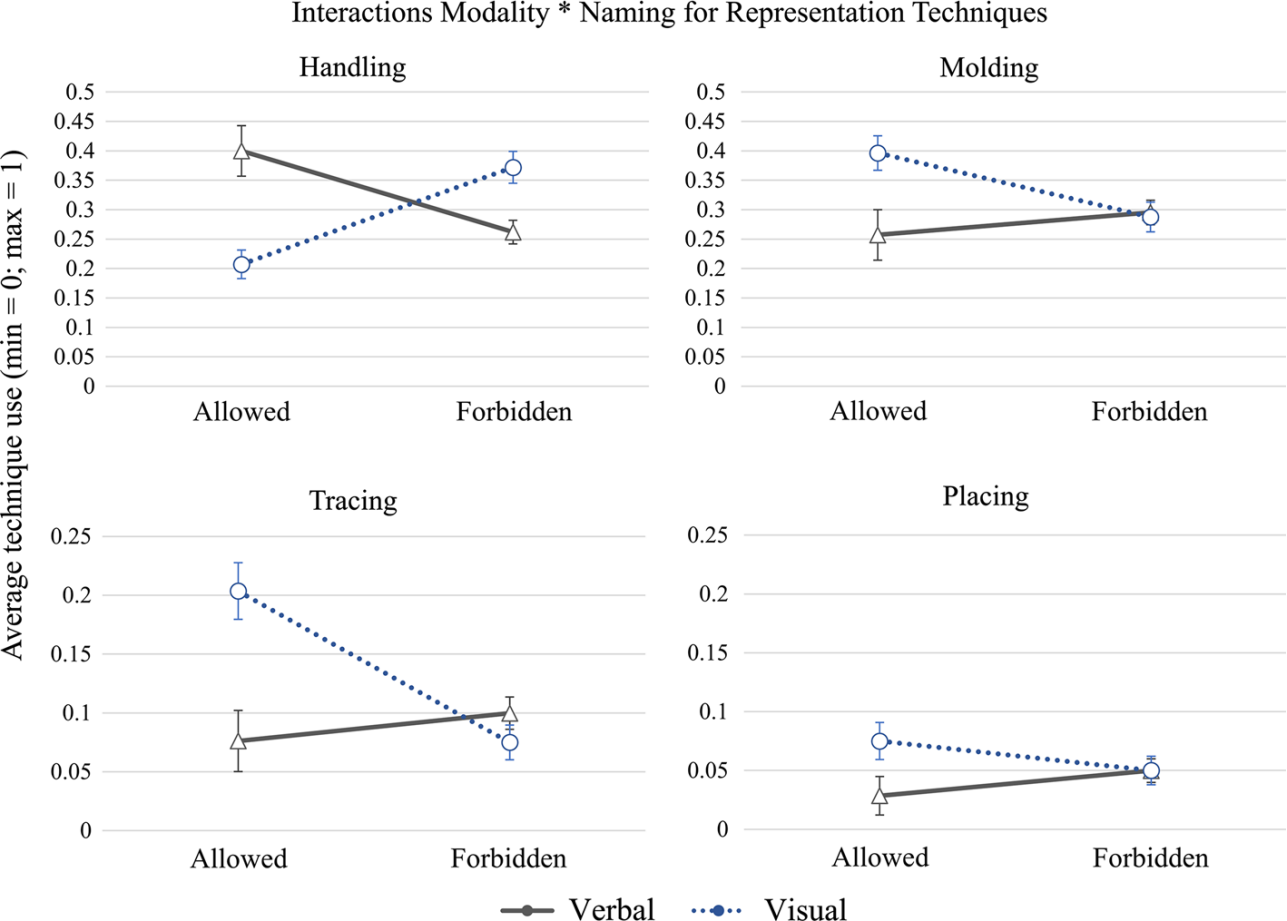
Interaction effects between modality and naming, for *handling*, *molding, tracing,* and *placing* gestures. The *error bars* indicate the standard error

There were no main effects of either *naming* or *modality*, but we found several interactions between the two (see Fig. 8). We find a crossover interaction between modality and naming for *handling* gestures (β = 2.12, *SE* = .51, *p* < .001). When naming was allowed, more handling gestures were produced in the verbal than in the visual condition. However, when naming was forbidden, the opposite pattern emerged, namely that speakers produced more handling gestures in the visual than in the verbal condition. For *molding* gestures, there was also a marginally generalizable interaction between naming and modality (β = −.92, *SE* = .49, *p* = .06), with speakers producing more molding gestures in the visual than in the verbal condition, when naming was allowed. Such a difference is not observed in the naming-forbidden condition. We find similar trends for other techniques that depict perceptual and structural properties of objects, such as *tracing* (β = −1.33, *SE* = .78, *p* = .08) and *placing* gestures (β = 1.33, *SE* = .56, *p* = .01) (see Fig. 8).

In sum, our results (1) highlight the preference for *handling* and *molding* representation techniques when depicting objects; (2) suggest that the technique used to represent an object is dependent of the degree of manipulability of the target object; and (3) show that speakers resort to different iconic strategies depending on the task at hand: With a simpler task (naming allowed), speakers depicted the shape of an object more often when they had seen a picture, and demonstrated the function of an object when they had read a word; with a more complex task (naming forbidden), these patterns disappeared.

## Discussion

In spontaneous talk, speakers integrate information from various sources into their communicative messages, prominently information from the immediately accessible visual environment acquired through the senses, and information that they retrieve from their own conceptualizations of objects and scenes. In this paper, we investigated how information acquired from these two sources influences the generation of co-speech gestures—particularly, how it influences the type of iconicity upon which these manual signs are constructed. To this aim, we designed a task in which speakers had to describe objects that differed in their manipulability degree to a peer based on either a word or a picture. In the first case, speakers would rely solely on their conceptual representation to generate a description, and in the second case, they would rely on a rich visual representation of the objects. Furthermore, we varied the complexity of the descriptions elicited, by allowing or forbidding speakers to explicitly name the target objects. We first discuss our results regarding gesture frequency, to then focus on the use of iconic strategies by speakers.

We expected speakers to gesture more in the visual condition regardless of task complexity, because speakers are faced with a higher volume of detail when looking at an image than when reading a word, much of this information being susceptible to be encoded in gesture (e.g., concrete information about shape, size, proportion, etc.). We argued that the lifelikeness and proximity evoked by pictures might render speakers prone to perceptual and motoric simulations which, in turn, may prompt gesturing, as proposed by Hostetter and Alibali (2008). Our results are partially compatible with this hypothesis. We found speakers to gesture more when describing an object presented as a picture than as a word, but this only happened when speakers were cued to produce simple descriptions.

One could interpret this pattern in terms of a cognitive difficulty continuum. It is likely that when describing became more complicated because speakers had to purposefully avoid naming the object, they resorted to a more deliberate strategy to circumvent the problem that may have overridden the effects of input modality. Past studies have shown that speakers tend to gesture more when a task is conceptually more challenging (e.g., Melinger and Kita 2007). In this light, our experimental conditions could be organized based on the difficulty they imposed on the speaker, ranging from the least complex (participants describing a word whilst being allowed to name it) to the most complex (participants describing a word whilst not being allowed to name it, and without having access to a visual representation). As it turns out, the easiest condition generated the lowest number of iconic gestures, and the most difficult condition elicited the most gesturing (recall Fig. 6). Importantly, this interaction between modality and task complexity was only found for iconic gestures, and it was not mirrored by either non-iconic gestures or by the number of words spoken by participants.

We think that these data reflect the richness of everyday interaction, as we compared gesture production in two frequent communicative scenarios, namely a scenario in which object naming was enough to complete a task and the use of gestures was less necessary, and a scenario where communicating required more elaboration. Our results suggest that gestures support speech differently when speakers performed more, and less, complex language tasks, with speakers adapting, and incrementally producing gestures, the more effort the task requires.

### The Use of Iconic Strategies

The mechanisms by means of which speakers select a particular iconic strategy during (spontaneous) gesturing, such as tracing a shape, remain poorly understood. By definition, co-speech gestures are idiosyncratic, in that they do not rely in codes and conventions like (sign) languages do (e.g., see Kendon’s continuum, McNeill 1992). Yet, recent studies have suggested that there might be some regularities in how speakers choose, and even combine iconic strategies while depicting concepts manually (e.g., Ortega and Özyurek 2016; Padden et al. 2015; van Nispen et al. 2014). Our study contributed to the existing body of literature in a number of ways.

First, our results revealed that *handling* and *molding* were the most frequently used iconic strategies to gesturally depict objects, and that the affordances of objects constrained the type of iconicity used—replicating findings by Masson-Carro and colleagues (2016). Although indirectly, this lends support to simulation-based accounts of gesture production (e.g., Hostetter and Alibali 2008), as both of these iconic strategies closely reflect direct interaction with objects, in the form of utilization (*handling*) and of haptic exploration (*molding*) of an invisible object that acts as the anchor for the movement. If gestures are indeed a window into our conceptualization of objects, our results suggest that action knowledge is prominent in the mind of the speaker, even during (non-goal-directed) communication tasks. Other iconic devices may not result from simulations of interaction with the described objects, but may be constructed upon, or recreate, graphic representations of such objects the way we create them on other media —as may be the case with *drawing*. Thus, iconic gestures seem to be constructed based on difference sources, one being action schemas containing knowledge on how to utilize an object and how that object may feel to the human hand, as well as action schemas related to the representation of knowledge on other media, which may be taken as a template to construct new gestures.

Secondly, our study showed that input modality and task complexity jointly—and independently of manipulability—influenced how speakers made use of iconicity in their gestures, with speakers being biased towards gesturally depicting action information when they described words, and towards gesturally depicting shape information when they described pictures, when naming of the objects was allowed. Pictures and words are very different representations, even when referring to the same objects. When speakers are asked to describe an image, they have access to more information than when they face a word. For example, an image representing a “toaster” also tells us whether it is old or new, whether it allows for one or multiple loaves of bread, whether it has a timer knob or a button, etc. Therefore, it is likely that a description based on an image will be guided by perceptually salient elements such as shape and color of the particular token displayed. When speakers need to describe a concept based on a word, they may still activate modal representations relative to an object’s shape or color, but it is likely that other elements such as function may become more salient given the communicative goal of the task. This prominence of function information when speakers access only their conceptual knowledge seems justified. A limited number of studies support the idea that character viewpoint gestures—which depict events from a first-person perspective, notably through enactments and imitative gestures (McNeill 1992; Parrill 2010)—are more informative to speakers (Beattie and Shovelton 2001, 2002). This is sensible from an information processing point of view, as the function of objects could be perceived as a more invariable and distinguishing attribute, and may lead to better object recognition than information about other visual cues such as shape, which often varies across tokens of the same object in the real world. Future research is needed to find out to what extent speakers think of particular iconic gestures as more communicative, and how they may use iconicity as a strategy to communicate more efficiently.

One important limitation of the present study is that we do not report analyses on the semantic content of speech. Some of the effects found in gesture may have been reflected in the words used by speakers as well. For instance, it could be that the increase in handling gestures while describing objects in the verbal, naming-allowed condition may have been accompanied by an increase in words describing function. Although this need not change our interpretation of the data, it raises the issue of at what point of the gesture generation process a particular iconic strategy to represent an object is selected. This is a question that is difficult to address within the current paradigm and should be targeted in a dedicated study, specifically designed to disentangle whether the iconic strategy chosen in gesture directly stems from a particular conceptual representation and whether (and how) it is mediated by speech production processes.

### Pinpointing the Effects of Manipulability

This study also has implications for how object affordances are processed. Earlier in this paper, we hypothesized that gestures may emerge from simulations of modal content and, as such, they inherit aspects of such simulations. Nevertheless, this could erroneously lead to the idea that the speaker herself has little control over the gestures she produces. We want to emphasize that we do not think this is the case. As this paper shows, producing simple references to objects resulted in gestures that took on aspects that were prominent in the modality the objects were presented in. Complex descriptions, on the other hand, may be exemplary of more strategically planned multimodal messages, constrained by the specific communicative pressures of the situation. Thus, it may be that either seeing or thinking of objects automatically activates several motor programs, and a regulatory mechanism is in charge to select among these obeying to constraints imposed by task and context (Borghi and Riggio 2015). This idea resonates well with what we observed in this study, with the modality effects disappearing when the task had added constraints (i.e., preventing speakers from naming the object). Still, the automaticity with which such a regulatory mechanism operates is not fully understood, and the same can be said for the choice of an iconic strategy in gesture. Gestures are assumed to be rather spontaneous, in the sense that speakers often seem unaware of having produced them. At the same time, previous research has shown that speakers seem to design the form of their gestures with their addressee’s in mind. For instance, Hoetjes et al. (2015) found that after cases of miscommunication with an addressee, speakers enhanced the form of the gestures they produced to succeed at meeting the task’s goals. Similarly, Campisi and Özyurek (2013) showed that speakers used more iconic gestures, and these were larger, when explaining how to prepare coffee to children—in comparison with to adults. We suggest that addressee adaptation may be reflected in speakers’ use of iconic strategies too, and we envision that similar paradigms to Hoetjes et al. (2015) or Campisi and Özyurek (2013) could be applied to study whether speakers tailor their use of specific representation techniques, and how deliberate or automatic this type of adaptation might be.

## Conclusion

In this study, we showed that the input modality (verbal, visual) matters in determining what aspects of a representation speakers will gesturally depict, but this is modulated by the complexity of the task. In addition, this study showed that the choice of an iconic strategy is dependent of the properties of the referent (i.e., its degree of manipulability) and, in general, highlighted a preference for *handling* and *molding* gestures, both of which closely reflect direct interaction with objects.

In the introduction to this paper, we argued that, in order to construct a manual sign that is evocative of a referent, a series of processes might be involved such as feature selection, schematization and the conversion into a motor program. Elucidating the relations among these processes is crucial so that we can implement the results into speech and gesture (both production and comprehension) models. A few questions that remain unexplored in this study, but that we think are crucial, relate to the nature of simulation—e.g., how could we gather direct empirical evidence to test whether gestures indeed stem from simulations of action?—as well as to the nature of iconic strategies: How are they exploited and combined by speakers in more complex situations? How do the different techniques stem from, and support thinking processes? How are different iconic devices processed, and comprehended by addressees? At what point(s) of the gesture generation process do the different constraints apply? We hope to address these issues in future research.

## Appendix 1

See Table 3.

**Table 3:** Results from the pre-test for all the included stimuli (translated to English)

Condition	Items (English)	Image manipulability	SD	Word manipulability	SD	Average manipulability	Word imageability %
High-manipulable	Mouse (PC)	94.74	15.29	96.06	9.9	95.4	97
High-manipulable	Toothbrush	94.55	9.09	96.24	12.12	95.395	100
High-manipulable	Pliers	93.58	14.85	96.7	7.78	95.14	100
High-manipulable	Scissors	93.61	15.43	95.39	10.14	94.5	97
High-manipulable	Screwdriver	92.87	11.03	95.76	8.7	94.315	97
High-manipulable	Brush	94.26	9.4	93.45	11.08	93.855	100
High-manipulable	Comb	93.19	18.93	93.76	14.4	93.475	100
High-manipulable	Calculator	93.77	15.84	93.03	12.16	93.4	97
High-manipulable	Paintroller	93.97	9.4	92.82	13.37	93.395	94
High-manipulable	Can opener	93.42	9.98	93.06	15.97	93.24	97
High-manipulable	Tennis racket	91.26	16.66	94.09	10.74	92.675	100
High-manipulable	Nut cracker	92.39	13.35	92.18	14.5	92.285	100
High-manipulable	Stapler	91.06	17.01	92.67	16.53	91.865	100
High-manipulable	Cheese slicer	91.16	16.56	91.91	16.78	91.535	94
High-manipulable	Keyboard	91.2	13.83	91.85	15.31	91.525	97
High-manipulable	Guitar	89.5	13.9	93.48	11.19	91.49	97
High-manipulable	Clothes pin	91.65	12.54	91.12	19.72	91.385	94
High-manipulable	Binoculars	90.23	14.95	91.79	16.55	91.01	97
High-manipulable	Bike pump	88.81	14.73	91.55	14.43	90.18	100
Low-manipulable	Bus	33.06	30.85	46.28	37.33	39.67	100
Low-manipulable	Cruise ship	18.81	27.04	25.03	31.84	21.92	100
Low-manipulable	Podium	46.55	30.68	38.58	33.85	42.565	84
Low-manipulable	Fireplace	44.65	33.34	20.63	29.91	32.64	84
Low-manipulable	Bird’s nest	41.29	35.94	37.88	34.4	39.585	94
Low-manipulable	Bathtub	52.13	32.19	48.61	38.24	50.37	94
Low-manipulable	Tombstone	29.81	28.08	31.31	30.51	30.56	100
Low-manipulable	Castle	19.45	28.14	21.03	26.52	20.24	100
Low-manipulable	Cross	42.55	32.75	47	32.4	44.775	94
Low-manipulable	Lamp post	29.65	29.89	27.79	29.44	28.72	97
Low-manipulable	Column	29.84	25.45	24.67	29.32	27.255	97
Low-manipulable	Radiator	44.52	29.03	49.42	35.66	46.97	94
Low-manipulable	Chimney	24.65	27.86	25.48	31.45	25.065	100
Low-manipulable	Shed	38.5	30.55	29.7	34.66	34.1	97
Low-manipulable	Toilet	51.29	34.41	48.03	36	49.66	100
Low-manipulable	Traffic light	32.81	29.39	31.06	32.84	31.935	100
Low-manipulable	Lighthouse	16.32	26.3	21.55	28.55	18.935	94
Animals	Tiger	10.06	18.17	14.91	24.14	12.485	100
Animals	Lion	8.48	17.18	17.27	26.01	12.875	94
Animals	Bear	4.55	8.16	22.52	30.61	13.535	94
Animals	Orca	12.26	22.92	16.27	28.15	14.265	94
Animals	Giraffe	10.74	17.62	23	30.33	16.87	94
Animals	Elephant	7.68	16.35	26.41	34.67	17.045	97
Animals	Fox	16.48	22.04	20.28	26.26	18.38	94
Animals	Kangaroo	14.58	18.16	23.25	29.07	18.915	97
Animals	Zebra	17.61	23.31	28.09	32.89	22.85	97
Animals	Owl	21.13	22.51	25.19	28.32	23.16	91
Animals	Squirrel	29.03	29.36	29.67	32.02	29.35	94
Animals	Ant	27.55	31.05	33.94	38.22	30.745	100
Animals	Pig	34.63	31.74	28.36	31.33	31.495	84

## Appendix 2: Task instructions administered to speakers (translated to English)

### Verbal condition, naming allowed

Thanks for your participation in this experiment.

After you press the spacebar, you will see a number of words referring to objects. You will describe these objects one by one, and you may mention the name of the object. Your task is to describe each of the objects in such a way that your partner understands what item you are describing. At the end of this experiment, your partner will complete a memory test. In this memory test, your partner will mark all items which he/she thinks you have described.

Press the spacebar to start with the first object, as well as to advance through the experiment.

### Verbal condition, naming forbidden

Thanks for your participation in this experiment.

After you press the spacebar, you will see a number of words referring to objects. You will describe these objects one by one, and you may not mention the name of the object. Your task is to describe each of the objects in such a way that your partner understands what item you are describing. At the end of this experiment, your partner will complete a memory test. In this memory test, your partner will mark all items which he/she thinks you have described.

Press the spacebar to start with the first object, as well as to advance through the experiment.

### Visual condition, naming allowed

Thanks for your participation in this experiment.

After you press the spacebar, you will see a number of images referring to objects. You will describe these objects one by one, and you may mention the name of the object. Your task is to describe each of the objects in such a way that your partner understands what item you are describing. At the end of this experiment, your partner will complete a memory test. In this memory test, your partner will mark all items which he/she thinks you have described.

Press the spacebar to start with the first object, as well as to advance through the experiment.

### Visual condition, naming forbidden

Thanks for your participation in this experiment.

After you press the spacebar, you will see a number of images referring to objects. You will describe these objects one by one, and you may not mention the name of the object. Your task is to describe each of the objects in such a way that your partner understands what item you are describing. At the end of this experiment, your partner will complete a memory test. In this memory test, your partner will mark all items which he/she thinks you have described.

Press the spacebar to start with the first object, as well as to advance through the experiment.

## Appendix 3

See Table 4.

**Table 4:** Means and Standard Deviations (SD) for the use of each representation technique under all experimental conditions

	Allowed		Forbidden	
	Verbal	Visual	Verbal	Visual
Enact				
Manipulable	0	0	.01 (.11)	.06 (.07)
Non manipulable	0	0	.02 (.15)	0.02 (.16)
Animals	0.14 (0.35)	.05 (.22)	.08 (.27)	.06 (.24)
Enclose				
Manipulable	.04 (.19)	.03 (.18)	.13 (.34)	.05 (.22)
Non manipulable	.24 (.43)	.05 (.23)	.14 (.35)	.09 (.28)
Animals	.19 (.4)	0.5 (.22)	.21 (.41)	.25 (.44)
Mould				
Manipulable	.04 (1.9)	.22 (.42)	.13 (.34)	.14 (.34)
Non manipulable	.48 (.51)	.53 (.5)	.47 (.5)	.47 (.5)
Animals	.42 (.5)	.42 (.51)	.47 (05)	.45 (.5)
Handle				
Manipulable	.8 (.4)	.49 (.5)	.49 (.5)	.64 (.48)
Non manipulable	.03 (.17)	.01 (.08)	.03 (.17)	.09 (.28)
Animals	0	0.05 (.21)	0	.01 (.12)
Place				
Manipulable	0	.01 (.09)	.03 (.16)	.02 (.15)
Non manipulable	.06 (.24)	.12 (.33)	.11 (.31)	.13 (.34)
Animals	.04 (.21)	.09 (.3)	.03 (.17)	.01 (.12)
Portray				
Manipulable	.11 (.32)	.06 (.24)	.09 (.29)	.08 (.27)
Non manipulable	.06 (.24)	.03 (.18)	.06 (.24)	.06 (.24)
Animals	0	.23 (.43)	.05 (.22)	.03 (.17)
Trace				
Manipulable	0	.18 (.39)	.08 (.28)	.04 (.19)
Non manipulable	.12 (.33)	.23 (.43)	.11 (.31)	.08 (.28)
Animals	.19 (.4)	.09 (.3)	.13 (.34)	.15 (.36)

## Footnotes

1 Note that, although we use words such as “select” or “choose”, we do not mean to imply that this is a deliberate process. We hope to address the nature of the selection process specifically in further research.

2 Note that, although written words or text are actually presented *visually*, we will refer to this condition throughout the paper as “verbal” condition, and use “visual” solely when referring to still or moving images.
